# Leveraging a Landscape-Level Monitoring and Assessment Program for Developing Resilient Shorelines throughout the Laurentian Great Lakes

**DOI:** 10.1007/s13157-019-01139-w

**Published:** 2019-04-25

**Authors:** Donald G. Uzarski, Douglas A. Wilcox, Valerie J. Brady, Matthew J. Cooper, Dennis A. Albert, Jan J. H. Ciborowski, Nicholas P. Danz, Anne Garwood, Joseph P. Gathman, Thomas M. Gehring, Greg P. Grabas, Robert W. Howe, Lucinda B. Johnson, Gary A. Lamberti, Ashley H. Moerke, Gerald J. Niemi, Todd Redder, Carl R. Ruetz, Alan D. Steinman, Douglas C. Tozer, T. Kevin O’Donnell

**Affiliations:** 1 Institute for Great Lakes Research, CMU Biological Station, and Department of Biology, Central Michigan University, Mt. Pleasant, MI, USA; 2 Department of Environmental Science and Ecology, SUNY College at Brockport, Brockport, NY, USA; 3 Natural Resources Research Institute, University of Minnesota Duluth, Duluth, MN, USA; 4 Present address: Burke Center for Freshwater Innovation, Northland College, Ashland, WI, USA; 5 Oregon State University, Corvallis, OR, USA; 6 University of Windsor, Windsor, ON, Canada; 7 University of Wisconsin-Superior, Superior, WI, USA; 8 Michigan Department of Environmental Quality, Lansing, MI, USA; 9 University of Wisconsin-River Falls, River Falls, WI, USA; 10 Environment and Climate Change Canada, Toronto, ON, Canada; 11 University of Wisconsin-Green Bay, Green Bay, WI, USA; 12 University of Notre Dame, Notre Dame, IN, USA; 13 Aquatic Research Laboratory, Lake Superior State University, Sault Ste. Marie, MI, USA; 14 LimnoTech Corporate HQ, Ann Arbor, MI, USA; 15 Annis Water Resources Institute, Grand Valley State University, Muskegon, MI, USA; 16 Long Point Waterfowl and Wetlands Research Program, Bird Studies Canada, Port Rowan, ON, Canada; 17 U. S. Environmental Protection Agency, Great Lakes National Program Office, Chicago, IL, USA

**Keywords:** The Laurentian Great Lakes, Coastal wetlands, Monitoring, Ecosystem health

## Abstract

Traditionally, ecosystem monitoring, conservation, and restoration have been conducted in a piecemeal manner at the local scale without regional landscape context. However, scientifically driven conservation and restoration decisions benefit greatly when they are based on regionally determined benchmarks and goals. Unfortunately, required data sets rarely exist for regionally important ecosystems. Because of early recognition of the extreme ecological importance of Laurentian Great Lakes coastal wetlands, and the extensive degradation that had already occurred, significant investments in coastal wetland research, protection, and restoration have been made in recent decades and continue today. Continued and refined assessment of wetland condition and trends, and the evaluation of restoration practices are all essential to ensuring the success of these investments. To provide wetland managers and decision makers throughout the Laurentian Great Lakes basin with the optimal tools and data needed to make scientifically-based decisions, our regional team of Great Lakes wetland scientists developed standardized methods and indicators used for assessing wetland condition. From a landscape perspective, at the Laurentian Great Lakes ecosystem scale, we established a stratified random-site-selection process to monitor birds, anurans, fish, macroinvertebrates, vegetation, and physicochemical conditions of coastal wetlands in the US and Canada. Monitoring of approximately 200 wetlands per year began in 2011 as the Great Lakes Coastal Wetland Monitoring Program. In this paper, we describe the development, delivery, and expected results of this ongoing international, multi-disciplinary, multi-stakeholder, landscape-scale monitoring program as a case example of successful application of landscape conservation design.

## Introduction

Coastal wetlands are critical components of the Laurentian Great Lakes ecosystem and have suffered extensive degradation and loss over the past two centuries (Snell 1987; Krieger et al. 1992; Schaefer 1994, Environment Canada 2002), and most have been greatly affected by land-use change and pollution (Bedford 1992; Wilcox 1995). Recognition and appreciation of the importance of coastal wetlands in the Laurentian Great Lakes ecosystem have grown markedly in recent decades as numerous important ecosystem functions have been ascribed to these habitats. For example, coastal wetlands provide critical breeding or migratory habitat for wildlife such as birds, mammals, reptiles, and amphibians (Herdendorf et al. 1981a, b, c, d, e, f; Mitsch and Gosselink 1993; Austen et al. 1994; Hecnar 2004; Hanowski et al. 2007a). These habitats are also critical spawning and nursery areas for many fish species of ecologic and economic importance (Herdendorf et al. 1981a; Chubb and Liston 1986; Klarer and Millie 1992; Jude et al. 2005). Additionally, coastal wetlands trap, process, and remove nutrients and sediment from Great Lakes nearshore waters, and their hydrologic effects on drainage patterns help recharge groundwater supplies (Burton 1985; Heath 1992). Accordingly, broad consensus has emerged among scientists, resource managers, and policy-makers, on the importance of coastal wetland functions to the entire Great Lakes ecosystem. However, over half of all Great Lakes coastal wetlands have been destroyed by human activities, and many remaining coastal wetlands suffer from anthropogenic stressors such as nutrient and sediment loading, fragmentation, invasive species, shoreline alteration, and water-level control (Burton 1985; Krieger et al. 1992; SOLEC 2007; Wilcox et al. 2008), as documented by a bi-national Great Lakes-wide mapping and attribution project (Albert and Simonson 2004; Ingram and Potter 2004). Therefore, conservation of remaining coastal wetlands and restoration of previously destroyed wetlands are vital components of restoring the Great Lakes ecosystem.

Conservation planning for wetlands requires sound science-based recommendations for managers to consider when setting priorities and making decisions. On a landscape scale, regional data utilizing a standardized methodology must be collected across an entire landscape to create a database for landscape-level conservation. For most regional-scale ecosystems, continual fluctuations in weather and ambient conditions require repeated sampling using standardized methods. Along the Laurentian Great Lakes shoreline, over 1000 coastal wetlands greater than four hectares in size with a surface water connection to the lake have been identified as critically important for habitat and maintenance of coastal processes, and thus, a program was designed and implemented to develop the necessary landscape-scale data sets for use in conservation and management decision-making.

Despite ongoing and often increasing threats to these ecosystems, no mechanism existed to determine the status and trends of Great Lakes coastal wetland condition at a basinwide scale. The Great Lakes Coastal Wetlands Consortium (GLCWC), consisting of U.S. and Canadian scientists, thus implemented the first-ever comprehensive, basin-wide Great Lakes Coastal Wetland Monitoring Program (GLCWMP), although an earlier U.S. Fish and Wildlife Service initiative provided the groundwork with Great Lakes-wide aerial-photo based mapping of all U.S. Great Lakes coastal wetlands (Herdendorf et al. 1981a, b, c, d, e, f). Our current program, funded under the Great Lakes Restoration Initiative by the U.S. Environmental Protection Agency, Great Lakes National Program Office (GLNPO), relies heavily on methodology previously developed, tested, and independently verified by GLCWC and other scientists. These standardized protocols efficiently and rigorously assess and report the condition of coastal wetlands basin-wide, and with repeated sampling, temporal trends in wetland condition for each of the Great Lakes are determined. This effort provides Great Lakes resource managers and decision-makers with the critical information on which to base strategic wetland protection, conservation, and restoration policies that will ultimately improve the condition of the Great Lakes ecosystem.

## GLCWMP Development

The monitoring program was established by many individuals from numerous organizations located around the Great Lakes basin. Nearly all of the original authors of the GLCWMP (GLCWC 2008), including many who contributed to indicator development and many of the lead scientists in a parallel effort, are principal investigators in this program.

### Site Selection

The pool of wetland sites used for selection was based on the GLCWC-GLNPO wetland coverage. Wetlands selected for sampling are four ha or larger, have a direct surface water connection to a Great Lake or connecting channel and are close enough to that lake or connecting channel to be influenced by it, and contain herbaceous or standing-water wetland vegetation zones. Previous basin-wide work by GLCWC indicated that smaller wetlands can be too small to sample, difficult to recognize, or ephemeral under certain hydrological conditions. Indicators and sampling protocols have not been evaluated for use in wooded wetlands because they are typically less influenced by Great Lake water-level fluctuations; therefore, these zones are not included in the GLCWMP.

The original coastal wetland map developed by GLCWC (Albert and Simonson 2004; Ingram and Potter 2004) contained more than 7000 wetlands. This map was analyzed by regional team experts, who identified 1034 coastal wetlands that were greater than four ha in size (greatlakeswetlands.org). We then developed an infrastructure for a long-term, statistically-sound monitoring framework, including the establishment of rotating panels of wetlands using a probabilistic selection process. The GLCWC (2008) developed this statistically-sound probabilistic design, recognizing that it is not likely that all Great Lakes coastal wetlands could be cost-effectively sampled in perpetuity. This framework allows for a statistically-valid prediction of overall Great Lakes coastal wetland condition based on a sub-set (one “panel”) of sites being sampled. We created a framework for coastal wetland condition assessment that is similar to the U.S. EPA Ecological Monitoring and Assessment Program (EMAP) for streams and lakes.

Our framework for probabilistic site selection was designed to be web-based. Site selection follows a stratified-random design based on: 1) wetland type (riverine, open coastal, barrier-protected); 2) region; and 3) Great Lake. The stratification process resulted in groups of sites for each lake (or lake x region, where lakes cross the regional boundary); sites were then randomized within each group. Each regional sampling team is assigned sites by random selection of one site from each group in each region and repeating this process to fill the roster of potential sites to be sampled during a given year. Teams over-select sites within each group to account for sites that fail to meet the minimum sampling criteria after onsite inspection. The group of sites where sampling is actually performed during year 1 forms the basis of a rotating panel design that ensures that all major wetlands will be sampled over a period of five years, with potential modification of this schedule based on the results of the first year of sampling. Finally, 10% of sites sampled each year are “re-sample” sites. These randomly-selected re-sample sites are chosen from sites that were sampled in the previous year. Re-sampling sites allows for the detection of wetland-by-year variability, or ‘trends’ in ecosystem health, as well as effects of water-level change on indicators. This stratified-random site-selection process ensures that the condition of Great Lakes coastal wetlands basin-wide can be inferred and statistically summarized based on the outcome of a single year’s sampling. In additional years, revisits to sites provide an indication of trends of ecosystem health basin wide. These predictions can then be re-evaluated with greater precision as additional sites are added to the sample pool.

In addition to the randomly-selected sites and the resampling sites, study crews also establish and sample a small number of “benchmark” sites for which there exist large amounts of historical data, represent extreme ends of the anthropogenic disturbance gradient, or are proposed/ongoing wetland restoration sites. Benchmark sites are sampled in the same manner as randomly-selected sites, but data from these sites are analyzed separately. Purposely selected sites are meant to ensure that sites meet the objective of sampling all wetland types across the full range of disturbance, which will aid indicator interpretation. Benchmark sites are chosen at locations where proposed restorations need to provide pre- or post-restoration implementation data, or where on-going restorations need post-restoration data (depending on the implementation of restoration activities at a site).

## Sampling Methodology

### Vegetation Community Indicators

Vegetation monitoring protocols (Uzarski et al. 2017) focus on 1) identifying and quantifying invasive plants that are considered indicators of degraded habitat (Albert and Minc 2004), 2) identifying significant interannual changes to submergent and floating-leaved vegetation, and 3) comparing local site mean Conservatism (mean C) values to regional mean C values (Herman et al. 2001). Mean C is based on two aspects, the plant’s dependability on specific habitat and its ability to tolerate stressors. Wetland and aquatic macrophytes are sampled at points along transects extending perpendicular to the shoreline at each site. Transects are selected to intersect major vegetation zones/types at each site, with three transects being established in each wetland. Once transects are established, endpoints are established using a handheld GPS. Sampling along transects perpendicular to the shoreline ensures that all vegetation types are represented because relatively distinct zones of wet meadow, emergent, and submergent vegetation occur from the upland toward open water in most coastal wetlands. This zonation is generally related to differences in water depth, wave energy, and the associated differences in substrate. At each sampling point, all plants are identified to species level, and areal coverage is estimated in 1 m × 1 m quadrats. Five quadrats are sampled in each zone, these are spaced equidistance apart on each of the three transects based on the size of the zone: wet meadow, emergent, and submergent. If a zone is too narrow to accommodate five quadrats, a perpendicular transect is placed at the mid-point of the zone for sampling of the five quadrats. Representative specimens of plants that cannot be identified in the field are returned to the laboratory and identified under magnification.

The Michigan Floristic Quality Assessment (FQA) program (Herman et al. 2001) is used to calculate mean C values. This technique is ideal for basin-wide vegetation assessment because it was designed for use in Michigan, which encompasses most of the latitudinal gradient encountered in the Great Lakes. Mean C for native species and total flora (including non-native species) are calculated for each wetland and compared to regional mean C values. For over 100 of the largest coastal wetlands across the Great Lakes, transect sampling results from earlier studies (Albert et al. 1987, 1988, 1989; Minc 1997; Minc and Albert 1998) are being used to compute Floristic Quality Index and mean C scores, which can be compared to scores from the present study to assist in the assessment of regional or local changes in wetland quality over the last 15 to 20 years.

### Invertebrate Community Indicators

The invertebrate monitoring protocol (GLCWC 2008; Uzarski et al. 2017) uses an Index of Biotic Integrity (IBI) for distinct plant zones based on the work of Burton et al. (1999) and Uzarski et al. (2004). The IBI metrics provide a comprehensive evaluation of how coastal wetland invertebrate communities vary along anthropogenic disturbance gradients. Because sample collection and metric calculations are based on specific vegetation zone types, which are ultimately determined by water depth and hydrology, the IBI is designed to accommodate inter-annual variations in water level (Uzarski et al. 2004). Although the macroinvertebrate-based IBI was developed specifically for fringing wetlands, the GLCWC recommended its use in all coastal wetland types. We continue to explore further metric development and refinement as new data are generated for riverine and barrier-protected wetlands.

Macroinvertebrate samples are collected using standard 0.5-mm mesh D-frame dip nets during July and August (Uzarski et al. 2004, 2017; GLCWC 2008). Three replicate samples, haphazardly spaced at least 20 m apart, are collected from up to five major plant zones in each wetland. Invertebrates are hand-picked from each sample in the field following the methods of Uzarski et al. (2004, 2017) and preserved in 70% ethanol. In the laboratory, organisms are identified to the lowest operational taxonomic unit necessary for IBI metric calculations (genus-level in most cases). Taxonomic keys such as Thorp and Covich (1991) and Merritt et al. (2008) are used for identification. Resulting data are used to generate IBI metric values as specified in the GLCWMP.

### Fish Community Indicators

Fish are key indicators of biotic integrity in streams (e.g., Karr et al. 1986; Lyons and Wang 1996) and to a lesser degree in lakes (e.g., Fabrizio et al. 1995; Whittier 1998). More recently, fish have been used to assess wetland condition. Recognition of the importance of coastal wetlands to Great Lakes fishes (e.g., Jude and Pappas 1992) initiated a movement toward using fishes as indicators of wetland health (Wilcox et al. 2002; Timmermans and Craigie 2003; Environment Canada and Central Lake Ontario Conservation Authority 2004; Uzarski et al. 2005; Cooper et al. 2018). Fish community-based indicators adopted by the GLCWC are a set of metrics that are combined to yield IBI scores for distinct plant zones (based on Uzarski et al. 2005 and Cooper et al. 2018). These fish-based IBIs were initially formulated for *Typha* (cattail) - and *Schoenoplectus* (bulrush) -dominated wetlands and were designed to be used in all five Great Lakes (Uzarski et al. 2005). Cooper et al. (2018) built on and expanded those to additional vegetation types. The IBIs were tested against multiple water quality and anthropogenic disturbance gradients. The fish-based IBIs were further tested and validated by Bhagat et al. (2007) in wetlands spanning all five Great Lakes.

Fish community sampling protocols (Uzarski et al. 2017) are conducted by setting three fyke nets in each plant zone, containing enough water to set the nets. Two sizes (large frame and small frame, all small mesh) are used depending on zone depth. The large frame nets are set where water depth is between 0.5 and 1 m, and the small frame nets are set in water less than 0.5 m (Uzarski et al. 2005). Nets are set adjacent to the major plant types or zones in each wetland, with leads extending into the area to be sampled (Uzarski et al. 2005; Cooper et al. 2007). Nets are set for one night (approximately 24 h), after which fish are collected, identified to species, counted, measured, and released alive. If positive species identification cannot be made in the field, voucher specimens are returned to the laboratory and identified. Fish data are used to calculate metrics that assess the health of a wetland and are based on major plant zones.

### Anuran and Bird Community Indicators

Over the past 30 years, considerable field data have been gathered and analyzed to develop anuran (frog and toad) and bird monitoring protocols in the Great Lakes region, especially in wetlands (Niemi 1980; Hanowski et al. 1990; Gibbs and Melvin 1993; Howe et al. 1998; Weeber and Vallianatos 2000; Price et al. 2004; Crewe and Timmermans 2005; Meyer et al. 2006; Hanowski et al. 2007a, b; Howe et al. 2007; Price et al. 2007; Etterson et al. 2009 and Tozer 2013, 2016)). This program builds on this existing work by 1) establishing a strategic baseline of site-specific data, and 2) articulating and validating a clear, scientifically rigorous plan for long-term monitoring of bird and anuran populations in Great Lakes coastal wetlands. The field component of this monitoring program uses the protocols contained in the GLCWMP, but additional data were collected to improve the protocols and to ensure compatibility with the existing volunteer Great Lakes Marsh Monitoring Program (GLMMP), a long-term volunteer citizen science program that monitors birds, anurans, and habitat at targeted sites within coastal and, to a greater extent, inland wetlands mainly throughout the southern portion of the Great Lakes basin (Tozer 2013, 2016; Tozer et al. 2018; Weeber and Vallianatos 2000; Meyer et al. 2006).

Specifically, we sample breeding anuran and bird populations using the GLCWMP protocol (Uzarski et al. 2017) in approximately 250 wetland sites per year. Data collection is coordinated as much as possible with the annual volunteer GLMMP (Tozer et al. 2017b, c). We also critically examine data to improve the biological, logistical, statistical, and monetary efficiency of the GLCWMP protocols. This involves assessing tradeoffs in representation, statistical power, and cost among different frequency and timing of sampling at the level of individual surveys, sampling points, and wetlands. For example, we have assessed tradeoffs among different numbers of sampling points per wetland (Hanowski et al. 2007b) and different durations of point counts (Tozer et al. 2017a).

For anurans, unlimited-distance point counts are used to identify presence and calling intensity of species within each wetland (Uzarski et al. 2017). Depending on wetland area, field samples for each wetland site consist of one to six survey points spaced at least 500 m apart. Sites are visited three times per breeding season during peak vocalization periods, with a minimum of 15 days between visits (unless inclement weather or other unforeseen circumstances intervene). Surveys are conducted between sunset and midnight and occur only during acceptable weather conditions (GLCWC 2008).

For birds, we use fixed-distance and (simultaneously) unlimited-distance counts at points located at least 250 m apart within each wetland habitat (Uzarski et al. 2017). Point-count surveys are conducted either from 30 min before sunrise to 4 h after sunrise or from 4 h before sunset to 30 min after sunset. The number of birds seen or heard is recorded during 15-min observation periods (5 min of passive observation, 5 min of broadcast calling, 5 min of passive observation) at each pointcount station (GLCWC 2008). Wetlands are surveyed at least twice per year, unless unforeseen circumstances prohibit return visits. Quantitative indicators have been developed from previous data gathered, as well as from this study. A number of approaches have been used and compared, including an Index of Biotic Integrity (Crewe and Timmermans 2005; GLCWC 2008) and a probability-based Indicator of Ecological Condition (Howe et al. 2007; Chin et al. 2015). Both methods are compatible with the field data-collection methods described above. Recommended indicators are selected based on their transparency and effectiveness in describing the ecological condition of Great Lakes coastal wetlands.

### Chemical/Physical Measurements

Basic chemical and physical data are collected concurrently with biological samples in accordance with the GLCWMP (GLCWC 2008). These covariate data represent important measures of wetland condition and are used to account for variability in the biotic indicators. Chemical/physical measurements are made in each vegetation type where fish and macroinvertebrate data are collected. Water samples were collected at mid-depth in acid-washed bottles for analysis of soluble reactive phosphorus (SRP), nitrate-N, ammonium-N, and alkalinity. Alkalinity is determined using titration of raw water samples with standardized sulfuric acid. Soluble reactive phosphorus, nitrate-N, and ammonium-N are measured using spectrophotometric methods (APHA 2005), automated when possible (e.g., Lachat system). Temperature, dissolved oxygen, chlorophyll *a*, oxidation-reduction potential, total dissolved solids, turbidity, pH, and specific conductance are measured in situ using a YSI, Hydrolab, or equivalent instrumentation. Instrument maintenance and calibration follow protocols recommended by the manufacturer and are standardized across participating laboratories. Physical habitat variables such as water depth and qualitative substrate composition estimates are also assessed at each sampling site. Parameters are ranked for each site relative to others and combined to form a disturbance gradient measure referred to as SumRank (Uzarski et al. 2005, 2017).

## Data Management System and Web Portal for Data Dissemination

The GLCWMP implemented a web-based data-entry system and portal (https://www.greatlakeswetlands.org) to support data management and dissemination needs associated with the program. The system generates data-entry web forms automatically from data structure specifications, typically based on field data sheets. This approach allows rapid implementation of required database structures. Because data entry forms are similar to field data sheets, field crews can enter data online quickly and efficiently, reducing data entry errors. We also built in simple checks on data, validating that values fall within expected ranges and forcing out-of-range values to be double-checked. Experience has shown that the use of drop-down menus built into the system greatly reduces the frequency of data entry errors, particularly for scientific names of taxa. The sample-type-specific data structures and site-level metadata defined in the Data Management System (DMS) described in GLCWC (2008) were used as a basis for this work. The DMS handles the metadata that accompany these data (e.g., methods, study design, field-data error codes, description of indicator calculations, etc.), which helps to ensure the system’s usefulness to future researchers, managers, and the public.

To accommodate requests for raw data, the DMS is able to export self-documenting data files that can be imported into standard statistical, spreadsheet, and database programs for analysis. A complete set of export files is automatically generated every night, and files can also be exported upon user request. In addition, the system has the capacity to calculate defined analyses on demand. The DMS calculates indicator values for all wetlands for which relevant data have been entered into the system. These integrated calculations also reduce calculation errors and ensure that all users are calculating indicators in the same manner.

In addition to providing access to raw monitoring datasets, the GLCWMP website provides a wetland site mapping tool that is specifically designed to support the needs of coastal managers and other stakeholders (https://www.greatlakeswetlands.org/map). The default view in the mapping tool displays the centroids and boundary delineations (i.e., polygons) for all sites monitored under the GLCWMP, and it provides site-specific information regarding hydrogeomorphic type and years sampled. Coastal managers and stakeholders also have access through this tool to IBI scores and species lists for vegetation, invertebrates, and fish, and are able to efficiently generate map views and export IBI scores. A complementary *Coastal Wetlands Decision Support Tool* (DST) (https://www.greatlakeswetlands.org/DST) has also been developed to provide further support for wetland site filtering, prioritization, and ranking for potential restoration and protection investments. The combined capabilities of these web-based, map-centric tools allow managers to track time trends for wetlands of particular interest or to compare wetlands to one another.

## Quality Assurance/Quality Control and Participant Training

Rigorous quality assurance/quality control (QA/QC) procedures are adhered to in all aspects of the monitoring program. QA/QC, both within and across collaborating laboratories, is essential to ensuring that the information collected is defensible and has quantified levels of precision and accuracy. Specific QA/QC protocols follow those recommended by U.S. EPA and the American Public Health Association (APHA 2005), as well as additional protocols developed specifically for this program (e.g., establishing standard taxonomic reference sets, taxonomy training requirements, cross-laboratory taxonomy validation). Fully-detailed methodology and QA/QC protocols were compiled into standard operating procedures (SOPs) that all members of the program team follow.

### Education and Outreach

After each field season, we have data to infer an unbiased estimate of wetland condition across the Great Lakes. These inferences are refined, updated, and expanded each year to disseminate information to managers, agency personnel, and the public. We work to ensure that personnel at local, state/provincial, non-governmental, and federal agencies are informed about (and trained in) the rigorous, cost-effective monitoring strategy that we developed.

This program offers a rare opportunity to obtain comprehensive and consistent data for coastal wetlands across the Great Lakes basin. We recognize the importance of disseminating monitoring results to program managers, policy makers and to the general public (greatlakeswetlands.org). In addition to coordinating with GLRI-funded information dissemination projects, regular communication with state, tribal, and provincial agencies has been established through formation of a Great Lakes Regional Wetland Monitoring Workgroup. This Workgroup is open to state and tribal resource agencies in EPA Region 5, with participation being extended to other Great Lakes states and provinces; GLC; the GLCWMP team and other wetland scientists; and federal resource agencies, including U.S. EPA, U.S. ACE, U.S. FWS, and NOAA. Michigan DEQ assumed responsibility for making contacts with state and federal resource agencies and other participants, along with organizing meetings.

The Workgroup encourages the active exchange of information among resource management agencies and the scientists who are gathering coastal wetland data, provides for accurate interpretation and dissemination of monitoring results to managers and decision makers, and assists program managers in integrating the results of this program with other major monitoring efforts in the Great Lakes region, including but not limited to the National Wetland Condition Assessment. The GLCWMP data management system (DMS) and web portal are linked from the GLNPO wetland monitoring webpage and is a publicly-accessible system essential for the long-term wetland monitoring program. Such a system allows access to all data collected, the indicator calculations, and summary information.

Training future Great Lakes researchers and state and federal agency personnel and resource managers is an important part of this program. Because we sampled every summer for five years in Phase I and continue an additional five years in Phase II (and beyond), we anticipate training dozens of graduate students, several post-doctorates, and dozens of under-graduate students who assist with field and lab work while learning about Great Lakes coastal wetland ecosystems and their importance. Students rarely have the opportunity to participate in projects of this scale. These students will be well-trained to become tomorrow’s Great Lakes leaders.

Finally, all other products developed by this program are transferred to GLNPO and posted on the project website. These include the monitoring plan, including lists of the rotating panels with wetlands to be sampled across years, the sampling protocols, standard operating procedures, Quality Assurance Project Plans (QAPPs), indicator calculations, refinements, new indicators, and final and interim reports, as well as all publications. Program leaders and PIs also present data and findings at regional and national conferences (e.g., International Association for Great Lakes Research, Society of Wetland Scientists) and in the peer-reviewed literature.

## Outcomes, Outputs, and Expected Results

The primary outputs of this program are 1) a comprehensive assessment of the overall condition of Great Lakes coastal wetlands, with summaries by individual Great Lake, ecoregion, and wetland type and 2) the implementation of a standardized, statistically-valid long-term monitoring program for coastal wetlands across the entire Great Lakes basin. These outputs lead to a better understanding of wetland condition and temporal trajectories by managers and agencies across the Great Lakes. These data and their summaries are critical to guiding the future outcomes for preventing further wetland degradation and loss through protection, conservation, and restoration (and tracking outcomes via this monitoring plan), and generally (and demonstrably) enhancing the condition of coastal wetlands treated as a whole. Secondary outputs that make this possible include 1) a database-management system consisting of a data-entry system (with metadata capabilities), data-export tools, indicator calculators and summary tools that allow the system to be used for years into the future to help monitor and assess the condition of coastal wetlands and communicate this information to the public, managers, agencies, researchers, and anyone else, 2) a standardized sample design with rotating panels of wetland sites to be sampled across years, accompanied by sampling protocols, QAPPs, and other methods documents, 3) background documents on the indicators, their development, and the calculations involved, and 4) all program reports and publications. All of these outputs are available on publicly-accessible websites.

**Fig. 1:**
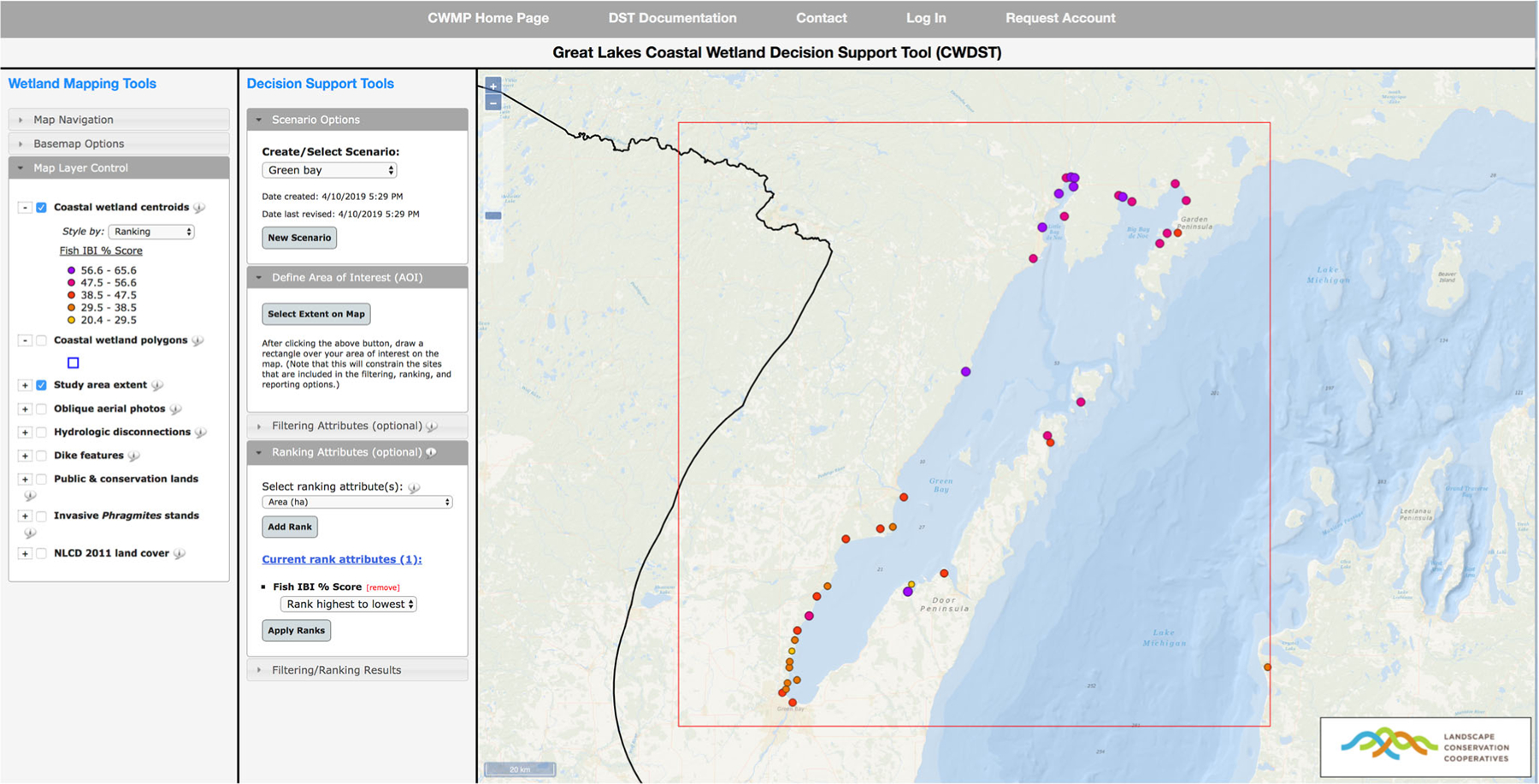
Great Lakes Coastal Wetland Decision Support Tool interface showing a ranking result based on Fish Index of Biotic Integrity scores for wetlands in Green Bay, Lake Michigan. Cooler colored dots indicate higher Fish IBI scores

These primary and secondary outputs have also allowed for development of the DST (www.greatlakeswetlands.org/DST) that can be used by managers to regionally rescale monitoring results regionally to address specific management questions. The DST is spatially flexible and can be implemented across scales from local (e.g., a few wetlands within a county or other management-relevant unit) to broader regional scales (e.g., wetlands within an entire state) or even the entire Great Lakes basin. Users interact with the DST using a map interface and a series of menus and dialogue boxes. The tool allows users to visualize monitoring results and other relevant information (e.g., wetland ownership, surrounding land use, etc.) within a geographic framework and to rank wetlands according to user-defined criteria associated with specific management objectives. Wetland selection and ranking schemes are constructed using attributes from five categories: 1) biotic condition (e.g., biotic community structure, indices of biotic integrity), 2) chemical and physical conditions (e.g., nutrient concentrations, dissolved ion concentrations, abiotic index of condition), 3) surrounding land use and human population density, 4) jurisdiction and ownership of wetland area, and 5) spatial habitat context of wetlands (e.g., proximity to other coastal wetlands, percentage of surrounding lands that are wetland or forest). By selecting a geographic region of interest and then filtering and ranking wetlands within that region using a customizable set of attributes, users can identify particular wetlands or subsets of wetlands of management interest (Fig. 1). The DST interface also provides various geospatial overlays such as surrounding land cover and land ownership maps, cover of invasive *Phragmites*, and oblique aerial imagery of each wetland, as well as data output functionality that is complimentary to the DMS download tool. Results from DST scenarios can be visualized on the map interface (Fig. 1) and/or downloaded with all relevant wetland attribute data.

Expected results of the GLCWMP include sampling of all major Great Lakes coastal wetland complexes across five-year time spans to provide data to assess the condition of these wetlands using indicators involving most major biotic groups, revision/refinement of wetland condition indicators and creation of new indicators, assessment of the effects of temporal variability on the various indicators, and identification of wetlands that can be used as regional reference sites. These results are being communicated via reports, presentations at regional and national meetings, the program website, Workgroup meetings, and publications. We also hold training workshops for technology transfer to agencies that may be taking over the routine wetland monitoring process.

While this program was developed specifically for work in coastal wetlands of the Laurentian Great Lakes, the framework is transferable to other ecosystem types located elsewhere in the world. For example, work was completed in 2014 to transfer the program to Poyang Lake, located in Jiangxi Province, China. Transferability to other ecosystems and locations is accomplished by first defining the targeted population of systems of interest. Once the geographic area and population of systems is defined, specific sub-types of ecosystems (e.g., major categories of wetlands or forests, etc.) have to be established to isolate natural variation among ecosystems based on sub-type, latitude, and climate. Sampling effort then has to be determined based on the number of system sites that can be sampled and how often sampling will occur. Time and resources are then allocated to reflect the number of sites of each type within a given sub-region based on latitude and climate (the strata). Once this stratified sampling design is established, resources can be allocated based on the number of sites within each strata. Indicators of ecosystem condition are then developed for each ecosystem sub-type by establishing reference sites that reflect the most pristine examples of each sub-type in each strata sub-category and comparing chemical and biological data from these sites to those from sites experiencing known anthropogenic disturbances. Once indicators are deemed trustworthy, a power analysis should be conducted to ensure that sampling effort is sufficient for each sub-type in each strata to detect changes in condition. As the size and scope of the program increases, the need for a robust QA/QC system escalates. Mid-season field sampling checks, sample exchanges, and sampling and identification refreshers, as well as field crew exchanges, are essential to ensure that comparable collections and sampling decisions are being made.

It is not necessary to transfer all parts of this sampling program to make use of the framework that we have established. For example, the SOPs for our sampling protocols have been adopted and adjusted for other ecosystems. The QA/QC system in particular can easily be adopted and applied to nearly any field sampling regime. The data management system, while tailored to specifically fit our types of samples, demonstrates how to set up a data management system for any particular set of protocols that can be accessed by many researchers across a widespread area, while still maintaining strong QA/QC. Our process for developing ecosystem condition metrics and IBIs can also be adapted to different ecosystem types. Chemical measures of water quality, similar to SumRank, are based on only annual sampling, but were developed to raise flags for extreme values. Extreme values for any region will indicate degraded vs reference systems.
